# Fabrication of Ciprofloxacin-Immobilized Calcium Phosphate Particles for Dental Drug Delivery

**DOI:** 10.3390/ma17092035

**Published:** 2024-04-26

**Authors:** Aniruddha Pal, Ayako Oyane, Tomoya Inose, Maki Nakamura, Erika Nishida, Hirofumi Miyaji

**Affiliations:** 1 Nanomaterials Research Institute, National Institute of Advanced Industrial Science and Technology (AIST), AIST Tsukuba Central 5, 1-1-1 Higashi, Tsukuba 305-8565, Japan; aniruddhapal8@gmail.com (A.P.); t.inose@aist.go.jp (T.I.); ma-ki-nakamura@aist.go.jp (M.N.); 2 Department of General Dentistry, Faculty of Dental Medicine, Hokkaido University, N13 W7 Kita-ku, Sapporo 060-8586, Japan; erikanishida@den.hokudai.ac.jp (E.N.); miyaji@den.hokudai.ac.jp (H.M.)

**Keywords:** coprecipitation, antibacterial activity, biofilm, carrier, ciprofloxacin

## Abstract

Calcium phosphate (CaP) particles immobilizing antibacterial agents have the potential to be used as dental disinfectants. In this study, we fabricated CaP particles with immobilized ciprofloxacin (CF), a commonly prescribed antibacterial agent, via a coprecipitation process using a supersaturated CaP solution. As the aging time in the coprecipitation process increased from 2 to 24 h, the CaP phase in the resulting particles transformed from amorphous to low-crystalline hydroxyapatite, and their Ca/P elemental ratio, yield, and CF content increased. Despite the higher CF content, the particles aged for 24 h displayed a slower release of CF in a physiological salt solution, most likely owing to their crystallized matrix (less soluble hydroxyapatite), than those aged for 2 h, whose matrix was amorphous CaP. Both particles exhibited antibacterial and antibiofilm activities along with an acid-neutralizing effect against the major oral bacteria, *Streptococcus mutans*, *Porphyromonas gingivalis*, and *Actinomyces naeslundii*, in a dose-dependent manner, although their dose–response relationship was slightly different. The aging time in the coprecipitation process was identified as a governing factor affecting the physicochemical properties of the resulting CF-immobilized CaP particles and their functionality as a dental disinfectant.

## 1. Introduction

Oral microorganisms such as bacteria, fungi, and viruses can cause oral diseases [1]. For example, certain types of oral bacteria, including *Streptococcus mutans* (*S. mutans*), *Porphyromonas gingivalis* (*P. gingivalis*), and *Actinomyces naeslundii* (*A. naeslundii*), are associated with two major oral diseases: dental caries and periodontal disease. *S. mutans* is the main component of dental plaque and the primary cause of caries [2]. The gram-negative bacterium *P. gingivalis* can cause periodontal disease, leading to tooth loss in the most severe case [3], whereas *A. naeslundii* is a major component of oral biofilms [4]. These bacteria often proliferate in narrow spaces in the mouth, such as dental fissures, pits, grooves, and periodontal pockets. Therefore, nano- and micro-carriers for local delivery of antibacterial agents are expected to be useful in controlling dental diseases caused by these bacteria [5,6].

Calcium phosphate (CaP) particles are one of the most promising carriers for dental drug delivery, because CaPs are chemically similar to the mineral fraction of human teeth [7,8], intrinsically safe, and white in color. Additionally, CaPs may promote remineralization of teeth by degrading into calcium and phosphate ions [9,10]. CaPs have long been used as biomaterials for dental care and restoration because of their good biocompatibility and osteoconductivity [11]. 

In the last few decades, CaP particles have been loaded with various antibacterial agents, such as silver nanoparticles [12], silver ions [13], gallium ions [14], zinc ions [15], chlorhexidine [16], and ciprofloxacin (CF) [17,18,19]. Among these agents, CF is particularly beneficial because it is a clinically approved fluoroquinolone antibiotic [20] that is white in color, exhibits high oral availability, and has antibacterial effects against a wide range of bacteria, including *S. mutans* [21,22], *P. gingivalis* [23,24], and *A. naeslundii* [25,26]. 

In previous studies, CF-immobilized CaP particles were fabricated by a coprecipitation process in a supersaturated CaP solution [18] or by an adsorption process [17,19]. The coprecipitation process can generally produce composite particles in which drugs are immobilized throughout the CaP matrix [27]. Hence, it is advantageous over the adsorption process in terms of having higher drug loading capacity [28]. CF-immobilized CaP particles prepared by the coprecipitation process showed antibacterial activity against certain types of bacteria [18]; however, their antibacterial activities against oral bacteria related to dental caries and periodontal disease have not been investigated.

In the previous coprecipitation process, the concentration of CF in the supersaturated CaP solution was varied to adjust the amount of CF immobilized in the CaP particles and to control their CF-release profile [18]. Here, we attempted a different approach based on the phase transformation of CaP to control the CF-release profile. We hypothesized that the aging time in the coprecipitation process would influence not only the amount of immobilized CF but also the CaP crystalline structure in the resulting CF-immobilized CaP particles, thereby affecting their CF-release profile. This hypothesis was based on previous results that showed that amorphous CaP particles changed into crystalline hydroxyapatite particles upon 24 h of aging during the coprecipitation process [29], and amorphous CaP particles released immobilized drugs faster than hydroxyapatite particles [30].

The first aim of the present study was to prepare CF-immobilized CaP particles with different crystalline phases and CF-release profiles by changing the aging time (2 h and 24 h) during the coprecipitation process. The second aim was to demonstrate the antibacterial and antibiofilm activities of the prepared particles against oral bacteria associated with dental caries and periodontal disease: *S. mutans*, *A. naeslundii*, and *P. gingivalis*.

## 2. Materials and Methods

### 2.1. Preparation of CF-Immobilized CaP Particles

We used four source solutions: calcium ion solution, phosphate ion solution, sodium carbonate solution, and CF solution. As calcium and phosphate ion solutions (500 mM for both), Calcium Chloride Corrective Injection 1 mEq/mL (Otsuka Pharmaceutical Co., Ltd., Tokyo, Japan) and Dibasic Potassium Phosphate Injection 20 mEq Kit (Terumo Corporation, Tokyo, Japan) were utilized, respectively. The sodium carbonate solution (500 mM) was prepared by adding sodium carbonate (FUJIFILM Wako Pure Chemical Corporation, Osaka, Japan) to ultrapure water in a glass vial, followed by sonication (VS-70RS1, AS ONE, Osaka, Japan) for a few minutes for complete dissolution. The CF solution (2 mg/mL) was prepared by dissolving CF (Fluorochem Ltd., Yokohama, Japan) in 0.0075 M HCl (FUJIFILM Wako Pure Chemical Corporation) via sonication for 5 min. Before preparing the supersaturated CaP solution, two solutions (solutions A and B) were prepared as described in a previous study [29]. Solution A was prepared by mixing the calcium ion solution (500 mM) and ultrapure water at a volume ratio of 4:21 in a 50 mL centrifuge tube. Solution B was prepared by mixing the phosphate ion solution (500 mM), sodium carbonate solution (500 mM), and ultrapure water at a volume ratio of 4:4:17 in a 50 mL centrifuge tube.

**Figure 1:**
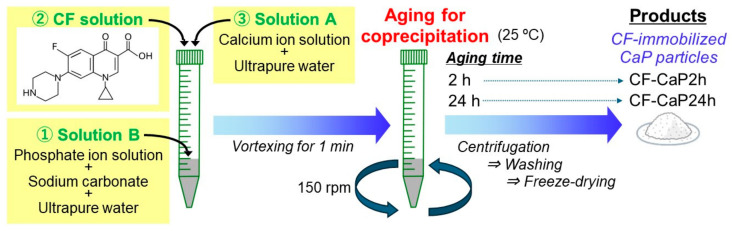
Schematic diagram showing the preparation of CF-immobilized CaP particles.

Finally, the supersaturated CaP solution was prepared by adding solution B (1 mL), followed by 2 mg/mL CF solution (1 mL), and lastly, solution A (2 mL) to a 15 mL centrifuge tube at 25 °C (Figure 1). Immediately after adding solution A, the final supersaturated CaP solution (4 mL) was vortexed for 1 min. Subsequently, the solution was aged under shaking at 150 rpm in a thermostatic shaker (M-BR-104P, TAITEC CORPORATION, Koshigaya, Japan) for 2 or 24 h at 25 °C to allow coprecipitation. The precipitate was collected by centrifugation (CN-1050, Hsiang Tai, New Taipei, Taiwan) at 6000 rpm for 5 min, followed by washing three times with ultrapure water. After washing, the product was resuspended in ultrapure water and freeze-dried for 24 h before further analysis. The resulting products were named CF-CaP2h and CF-CaP24h according to the aging times of 2 and 24 h, respectively.

### 2.2. Characterization of the Products

The morphologies of the products were examined using field-emission scanning electron microscopy (FESEM; SU8020, Hitachi High-Tech Corporation, Tokyo, Japan). The products were sputter-coated with gold for 2 min using a sputter-coating machine (SC-701MkII, Sanyu Electron Co., Ltd., Tokyo, Japan) before FESEM analysis. The chemical compositions of the products were examined without coating using an energy-dispersive X-ray (EDX) spectrometer (AZtecOne, Oxford Instruments plc, Abingdon, UK) equipped in a tabletop SEM (TM4000Plus II, Hitachi High-Tech Corp.). Before the EDX analysis, the products were mounted on a silicon sample holder using carbon tape.

The nanostructures of the products were further investigated using a transmission electron microscope (TEM; JEM-2100, JEOL Ltd., Tokyo, Japan) operating at 200 kV. Prior to TEM analysis, the products were mounted on a formvar-supported copper grid (JEOL Ltd.) and dried under reduced pressure. The diameter of the product was determined from 100 particles in the TEM images using ImageJ software (ver. 1.54).

The crystalline structure of the products was investigated by X-ray diffractometry (XRD; Rigaku RINT-Ultima III, Tokyo, Japan) with CuKα radiation (λ = 0.154 nm) at 40 kV and 30 mA. The chemical bonds of the products were investigated using a Fourier-transform infrared (FTIR) spectrometer (FT/IR-4700, JASCO Corporation, Hachioji, Japan) equipped with an attenuated total reflection accessory and a monolithic diamond crystal.

The Ca and P contents of the products were determined by performing inductively coupled plasma optical emission spectrometry (ICP-OES; ULTIMA2, HORIBA, Ltd., Kyoto, Japan). Before the ICP-OES analysis, the products were dissolved in 0.1 mL of 6 M HCl solution (FUJIFILM Wako Pure Chemical Corporation), which was subsequently diluted with 9.9 mL of ultrapure water.

### 2.3. Determination of CF-Immobilization Efficiency

The immobilization efficiency of CF in the products was calculated by dividing the CF amount in the product by the total amount of CF (2.0 mg) added to the supersaturated CaP solution in the same tube, using the following equation.

$$\mathrm{Immobilization}\;\mathrm{efficiency}\;\mathrm{of}\;\mathrm{CF}\;(\%)=100\times \frac{CF\;amount\;in\;the\;product\;(mg)}{CF\;amount\;in\;the\;solution\;(mg)}$$

**Figure 2:**
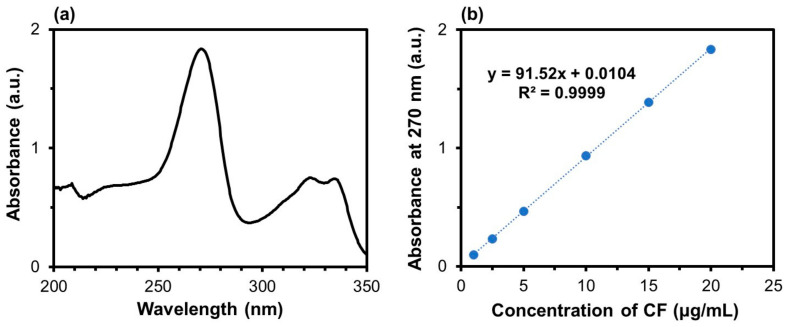
(**a**) UV absorption spectrum of the 20 μg/mL CF solution and (**b**) absorbance of the CF solutions with various concentrations at 270 nm.

The CF amount in the product was determined as follows: After the first centrifugation following the coprecipitation process (Section 2.1), the supernatant was taken out from the tube and diluted 40 times with a physiological salt solution (pH 7.4 at 37 °C). The physiological salt solution was prepared according to a previous report [31]. The amount of CF in the diluted supernatant was determined using a UV–visible spectrophotometer (UV-2450, SHIMADZU CORPORATION, Kyoto, Japan). For measurements, 1 mL diluted supernatant was poured in a UV-transparent disposable cuvette (BrandTech^®^ 759210, BrandTech Scientific, Essex, MA, USA), and the absorbance was measured at 270 nm, which correspond to the maximum absorption wavelength (Figure 2a). Standard solutions with various CF concentrations were prepared by diluting the 2 mg/mL CF solution described in Section 2.1 with the physiological salt solution. The absorbance of these solutions was then measured at 270 nm to obtain a calibration curve (Figure 2b). The amount of CF in the product was calculated by subtracting the amount of residual CF in the supernatant from the total amount of CF added to the supersaturated CaP solution. Six independent batches were used to obtain average and standard deviation (SD) values.

### 2.4. CF-Release Assay

The release of CF from the product was studied in the physiological salt solution (pH 7.4 at 37 °C). First, the product was suspended in the appropriate amount of ultrapure water such that the concentration of CF in the suspension was 0.40 mg/mL. The suspension (0.5 mL) was poured into a dialysis tube (Bio-Tech MWCO 12000, Bio-Tech, Taoyuan, Taiwan), which was kept in 10 mL of physiological salt solution in a 25 mL tube. After incubation at 37 °C for 0, 1, 3, 5, 7, 24, 48, and 72 h, an aliquot of 1.0 mL was sampled from the physiological salt solution, and fresh physiological salt solution (1.0 mL) was added to it. The amount of CF released into the physiological salt solution was determined by measuring the absorbance at 270 nm using the UV–visible spectrophotometer as described in Section 2.3. The percentage of CF released into the physiological salt solution from the net dose of CF (0.2 mg) added to the dialysis tube was calculated using the following equation.

$$\mathrm{Percentage}\;\mathrm{of}\;\mathrm{CF}\;\mathrm{released}\;(\%)=100\times \frac{CF\;amount\;in\;the\;physiological\;salt\;solution\;(mg)}{CF\;dose\;added\;to\;the\;dialysis\;tube\;(mg)}$$

To investigate the diffusion of free CF through the dialysis tube, 0.40 mg/mL CF solution (0.5 mL) was poured into the dialysis tube and tested using the same procedure. Three independent batches were used to determine the average and SD values.

### 2.5. Antibacterial Assay

The antibacterial properties of the products were assayed against *S. mutans*, *A. naeslundii*, and *P. gingivalis*. Each bacterium was procured from the American Type Culture Collection (Manassas, VA, USA) and frozen until further analysis. First, the frozen bacterial stocks were thawed and grown on brain heart infusion (BHI) medium (Pearlcore^®^, Eiken Chemical, Co., Ltd., Tokyo, Japan) supplemented with 0.1% antibiotic (0.05% gramicidin D and 0.05% bacitracin, FUJIFILM Wako Pure Chemical Corporation) and 1% sucrose (FUJIFILM Wako Pure Chemical Corporation). 

In a 96-well plate, *S. mutans* (1.1 × 10^6^ CFU/200 µL/well), *A. naeslundii* (1.0 × 10^6^ CFU/200 µL/well), and *P. gingivalis* (2.8 × 10^9^ CFU/200 µL/well) were incubated for 24 h at 37 °C under anaerobic conditions in the BHI medium supplemented with the product at various doses: 0 (ctrl), 0.001, 0.01, and 0.1 *w*/*v*%. After incubation, the metabolic activity of the bacteria (proportional to the number of living bacteria) was assayed using a microbial viability assay kit-WST (DOJINDO Laboratories, Mashiki, Japan) according to the manufacturer’s instructions, and the absorbance was measured at 450 nm using a microplate reader (Multiskan FC, Thermo Fisher Scientific, Waltham, MA, USA). The pH of each bacterial suspension was measured before and after incubation using a portable pH meter (LAQUA-PH-SE, HORIBA, Ltd., Kyoto, Japan).

### 2.6. Biofilm Formation Assay

The antibiofilm activity of the products was assayed against *S. mutans*. Biofilms were created on peg lids of a biofilm formation assay kit (DOJINDO Laboratories) by incubating *S. mutans* (5.5 × 10^6^ CFU/200 µL/well) for 24 h. Subsequently, the biofilms were incubated anaerobically for 48 h in the BHI medium supplemented with the product at various doses: 0 (ctrl), 0.001, 0.01, and 0.1 *w*/*v*%. After incubation, the relative amount of biofilm was assayed using a biofilm formation assay kit according to the manufacturer’s instructions, and the absorbance was measured at 595 nm using a microplate reader. In addition, the metabolic activity of the bacteria in the biofilm was assayed using a biofilm viability assay kit (DOJINDO Laboratories) according to the manufacturer’s instructions by measuring the absorbance at 450 nm using the microplate reader.

### 2.7. Statistical Analysis

For the antibacterial and antibiofilm assays, six wells were used for each condition to determine average and SD values. One-way analysis of variance (ANOVA) and Tukey’s post hoc multiple-comparison test were used to determine the differences between the average values of the groups. Statistical significance was set at *p* < 0.05.

## 3. Results

### 3.1. Morphological Analysis

**Figure 3:**
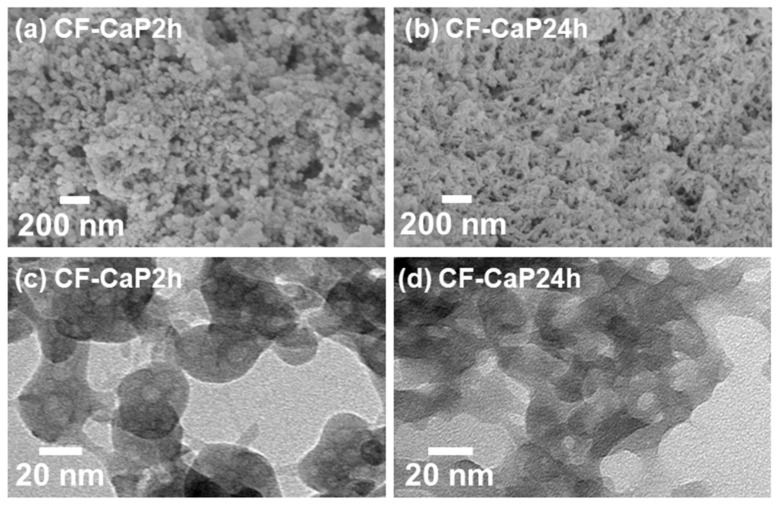
(**a**,**b**) FESEM and (**c**,**d**) TEM images of (**a**,**c**) CF-CaP2h and (**b**,**d**) CF-CaP24h.

The two products, CF-CaP2h and CF-CaP24h, were particles with different morphologies. As shown in the FESEM and TEM images (Figure 3a,c), CF-CaP2h consisted of nearly spherical particles with a primary particle diameter of ~30 nm. In contrast, CF-CaP24h consisted of irregularly shaped particles with indistinct boundaries (Figure 3b,d).

### 3.2. Chemical Analysis

**Figure 4:**
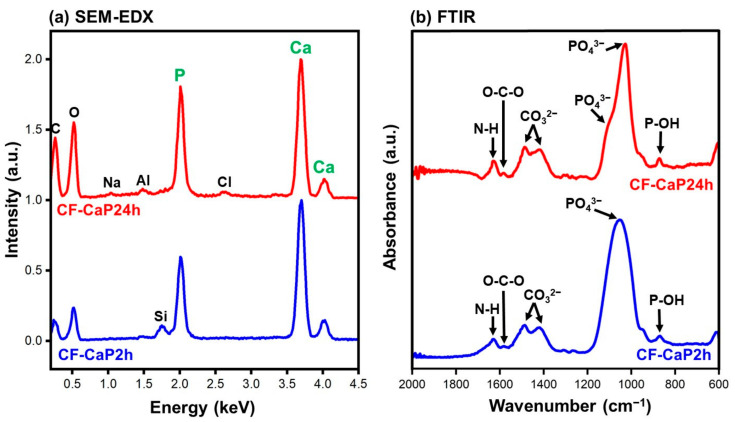
(**a**) SEM-EDX and (**b**) FTIR spectra of CF-CaP2h and CF-CaP24h. The peaks of Al and Si in (**a**) are from the sample holders.

Both particles, CF-CaP2h and CF-CaP24h, were composed of CaP and CF. In the SEM-EDX spectra (Figure 4a), strong peaks of Ca and P were detected in both CF-CaP2h and CF-CaP24h, indicating that these particles mainly consisted of CaP. Peaks of nitrogen or fluoride, which are component elements specific to CF, were not detected in the SEM-EDX spectra. On the other hand, in the FTIR spectra (Figure 4b), peaks of O-C-O asymmetric vibration (1585 cm^−1^) [32] and N-H bending vibration (1615 cm^−1^) [33], ascribed to CF, were detected in both CF-CaP2h and CF-CaP24h in addition to the distinctive peaks ascribed to CaP: P-OH stretching vibration (872 cm^−1^) [34], PO_4_^3−^ stretching vibration (1029–1051 cm^−1^) [35], and CO_3_^2−^ asymmetric stretching (1483 and 1413 cm^−1^) [36]. This suggested the presence of CF in both CaP particles.

**Figure 5:**
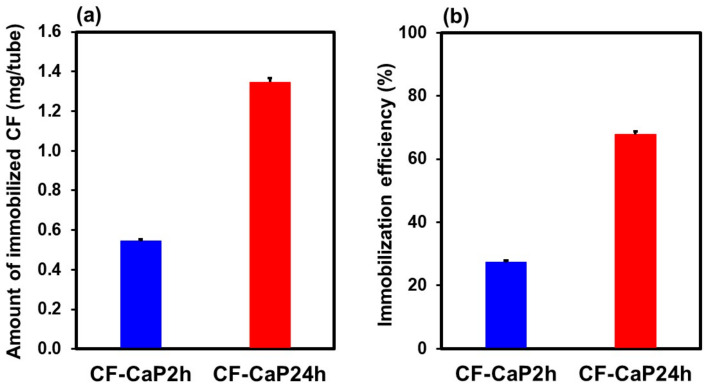
(**a**) Amount of immobilized CF and (**b**) immobilization efficiency of CF in CF-CaP2h and CF-CaP24h (n = 6, average + SD).

The CF-immobilization efficiency, amount (mg/tube) and content (*w*/*w*%) of the immobilized CF, and yield of the particles were greater for CF-CaP24h than that for CF-CaP2h. The amount of CF immobilized in the CaP particles was 2.5-fold greater for CF-CaP24h (1.35 ± 0.02 mg) than that for CF-CaP2h (0.54 ± 0.01 mg), as shown in Figure 5a. Since the yields of particles obtained from the supersaturated CaP solution (per one tube) were 12.9 mg and 15.8 mg for CF-CaP2h and CF-CaP24h, respectively, the CF content in the particles was 4.22 ± 0.09 and 8.48 ± 0.14 *w*/*w*% for CF-CaP2h and CF-CaP24h, respectively. From the amount of immobilized CF, the immobilization efficiencies of CF were calculated as 27.3 ± 0.5 and 67.8 ± 1.0% for CF-CaP2h and CF-CaP24h, respectively (Figure 5b).

### 3.3. Crystalline Structural Analysis

**Figure 6:**
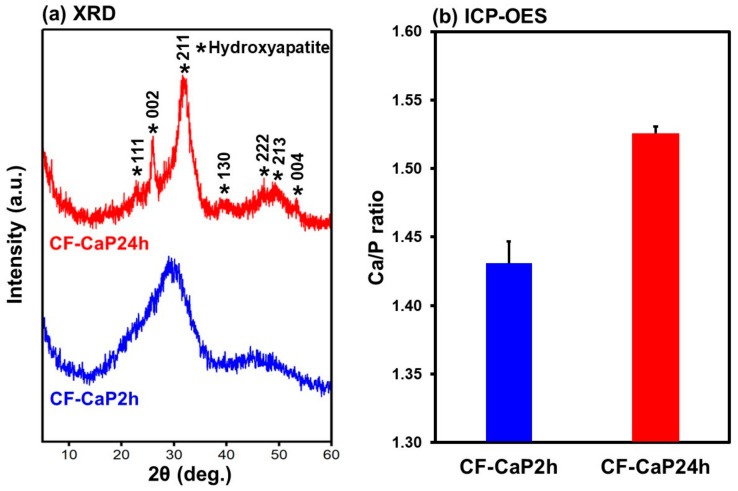
(**a**) XRD patterns and (**b**) Ca/P elemental ratios of CF-CaP2h and CF-CaP24h (n = 3, average + SD).

The CaP phase in CF-CaP2h was amorphous, whereas that in CF-CaP24h was Ca-deficient, low-crystalline hydroxyapatite. In the XRD pattern of CF-CaP2h (Figure 6a), a broad peak was observed at approximately 30°, which was attributed to CaP in the amorphous phase [37]. No other peaks ascribed to crystalline CaP phases were detected. In contrast, CF-CaP24h exhibited peaks ascribed to low-crystalline hydroxyapatite (Figure 6a). Based on the ICP-OES result (Figure 6b), the Ca/P elemental ratio of CF-CaP2h was 1.43 ± 0.02, which increased to 1.53 ± 0.01 for CF-CaP24h, most likely due to the amorphous-to-crystalline transformation in the CaP phase by prolonged aging [37]. The Ca/P elemental ratio of CF-CaP24h was lower than that of stoichiometric hydroxyapatite (1.67), indicating that the CaP phase in CF-CaP24h was Ca-deficient hydroxyapatite. As shown in Figure 4b, a single broad peak of PO_4_^3−^ stretching vibration (1051 cm^−1^) in CF-CaP2h was split into a sharp, strong peak (1029 cm^−1^) and a shoulder peak (1109 cm^−1^) in CF-CaP24h, which is a phenomenon observed for the phase transformation from amorphous CaP to hydroxyapatite [38,39]. These results revealed that the amorphous CaP phase in CF-CaP2h was converted to a crystalline hydroxyapatite phase in CF-CaP24h during prolonged aging (from 2 to 24 h). The Ca/P elemental ratio of hydroxyapatite precipitated from aqueous solutions increases up to nearly 1.67 through maturation with increasing aging time. Considering the relatively low Ca/P elemental ratio of CF-CaP24h, it most probably still contained residual amorphous phase and would have therefore undergone further maturation if aged for more than 24 h.

### 3.4. CF-Release Assay

**Figure 7:**
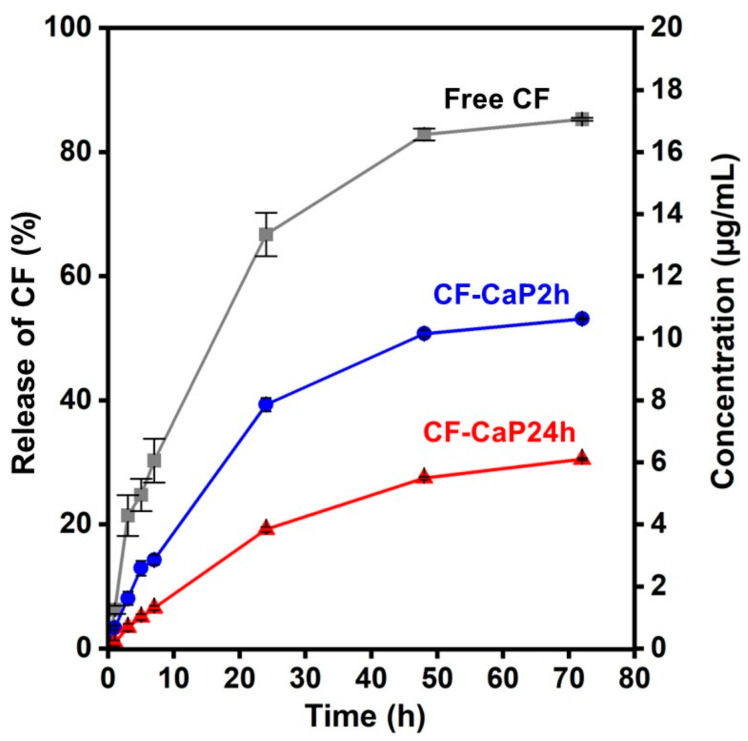
Changes in the percentage of CF detected in the physiological salt solution among the net CF dose added to the dialysis tube (left axis) and CF concentration (right axis) of the physiological salt solution during incubation of CF-CaP2h (blue circle), CF-CaP24h (red triangle), and free CF (grey square) in the dialysis tube for up to 72 h (n = 3, average ± SD).

CF-CaP24h allowed slower CF release than CF-CaP2h in the physiological salt solution. Figure 7 shows the release profiles of CF (percentage and concentration) from CF-CaP2h and CF-CaP24h during 72 h incubation. In this assay, the suspension of particles (net CF concentration, 0.40 mg/mL) stored in the dialysis tube was kept in the physiological salt solution to separate the particles from the test solution (physiological salt solution). For both CF-CaP2h and CF-CaP24h, the concentration of CF in the physiological salt solution increased over time. Because particles (larger than 30 nm) cannot pass through the dialysis tube (pore size of ~2.5 nm), the CF detected in the physiological salt solution was derived from the CF released from CF-CaP2h and CF-CaP24h. Free CF showed a faster increase in the CF concentration in the physiological salt solution (Figure 7). This indicates that the diffusion of CF through the dialysis tube was not the rate-determining step in the CF-release assay for CF-CaP2h and CF-CaP24h. Thus, the rate of increase in the CF concentration of the physiological salt solution reflects the CF-release rate from these particles. In other words, the CF release was faster for CF-CaP2h than for CF-CaP24h. This difference between CF-CaP2h and CF-CaP24h was likely caused by the different crystalline structures of their CaP matrices.

### 3.5. Antibacterial Assay

**Figure 8:**
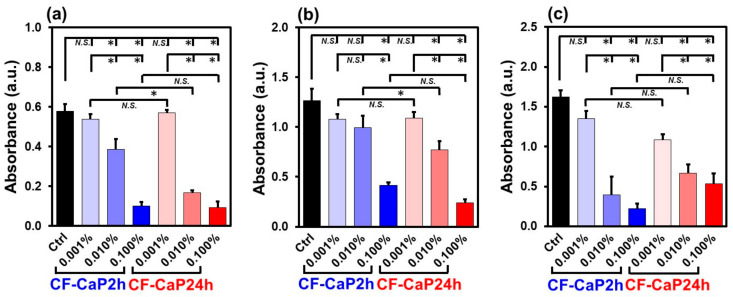
Relative number of living bacteria (absorbance at 450 nm) after the incubation of (**a**) *S. mutans*, (**b**) *A. naeslundii*, and (**c**) *P. gingivalis* in the presence of CF-CaP2h and CF-CaP24h at various concentrations: 0 (Ctrl), 0.001, 0.01, and 0.1 *w*/*v*% (n = 6, average + SD, * *p* < 0.05, *N.S.*: not significant).

Both CF-CaP2h and CF-CaP24h showed dose-dependent antibacterial activity against all the tested bacteria. At a lower dose (0.001 *w*/*v*%), neither CF-CaP2h nor CF-CaP24h exhibited antibacterial activity against any tested bacteria, as shown in Figure 8. At a medium dose (0.01 *w*/*v*%), both particles showed antibacterial activity against all tested bacteria except for *A. naeslundii* (only CF-CaP24h was effective). At a higher dose (0.1 *w*/*v*%), both particles showed antibacterial activity against all tested bacteria. CF-CaP24h exhibited stronger antibacterial activity than CF-CaP2h against *S. mutans* (Figure 8a) and *A. naeslundii* (Figure 8b) at a medium dose (0.01 *w*/*v*%). Against *P. gingivalis*, the opposite trend (stronger for CF-CaP2h) was observed at medium and higher doses (0.01 and 0.1 *w*/*v*%), although the difference between the two particles was not statistically significant (Figure 8c).

**Figure 9:**
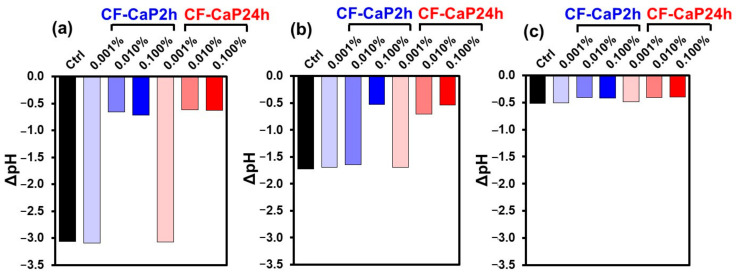
pH changes in suspensions of (**a**) *S. mutans*, (**b**) *A. naeslundii*, and (**c**) *P. gingivalis* after incubation for 24 h in the presence of CF-CaP2h and CF-CaP24h at various doses: 0 (Ctrl), 0.001, 0.01, and 0.1 *w*/*v*%.

In the assay with *S. mutans* and *A. naeslundii*, the pH of the bacterial suspension decreased, which was alleviated by higher doses of CF-CaP2h and CF-CaP24h. Figure 9 shows the changes in the pH of the bacterial suspensions after 24 h incubation in the presence of various doses of CF-CaP2h and CF-CaP24h. The pH value of the *S. mutans* suspension decreased by 3.1 after incubation in the absence of the particles (Ctrl), whereas the decrease in pH was less than 0.8 in the presence of higher doses (0.01 and 0.1 *w*/*v*%) of CF-CaP2h and CF-CaP24h (Figure 9a). This indicated that these particles had an acid-neutralizing effect [40]. A similar effect was observed for *A. naeslundii*; the decrease in pH (1.7) of the *A. naeslundii* suspension during incubation was reduced to 0.5 in the presence of a higher dose (0.1 *w*/*v*%) of CF-CaP2h and CF-CaP24h (Figure 9b). At a medium dose (0.01 *w*/*v*%), only CF-CaP24h exerted an acid-neutralizing effect, and CF-CaP2h had no apparent effect on the pH of the bacterial suspension (*A. naeslundii*). For *P. gingivalis*, only a slight decrease in the pH of the bacterial suspension was observed, irrespective of the presence (at any concentration) or absence of the particles (Figure 9c).

### 3.6. Biofilm Formation Assay

**Figure 10:**
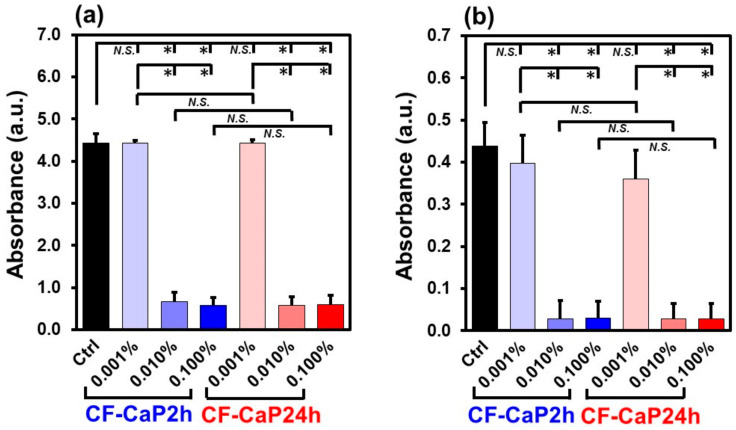
(**a**) Relative amounts of biofilms (absorbance at 595 nm) and (**b**) relative metabolic activity of the bacterial (*S. mutans*) biofilm (absorbance at 450 nm), after incubation of *S. mutans* in the presence of CF-CaP2h and CF-CaP24h at various doses: 0 (Ctrl), 0.001, 0.01, and 0.1 *w*/*v*% (n = 6, average + SD, * *p* < 0.05, *N.S.*: not significant).

Both CF-CaP2h and CF-CaP24h inhibited the biofilm formation at higher doses. In this study, we performed two assays using biofilms formed by *S. mutans*. Both biofilm formation (Figure 10a) and the metabolic activity of the bacterial biofilm (Figure 10b) were inhibited in the presence of CF-CaP2h and CF-CaP24h at higher doses of 0.01 and 0.1 *w*/*v*%.

## 4. Discussion

**Figure 11:**
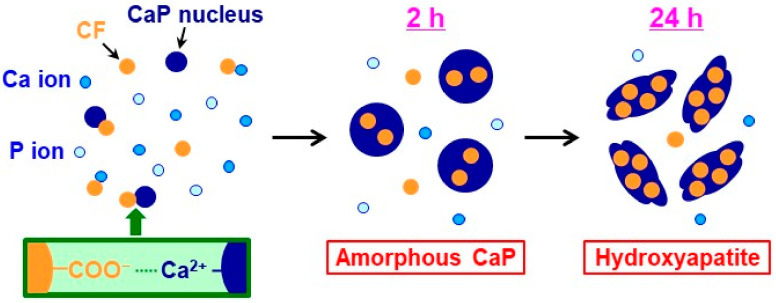
Schematic showing the formation of CF-immobilized CaP particles in the supersaturated CaP solution.

CF-immobilized CaP particles with different crystalline phases were successfully prepared by the coprecipitation process in the highly supersaturated CaP solution supplemented with CF by varying the aging time (2 and 24 h). The probable mechanism for the formation of the CF-immobilized CaP particles is as follows (Figure 11): First, homogeneous nucleation of CaP (amorphous phase nucleation) occurred in the supersaturated CaP solution, and the nuclei grew into nearly spherical CaP particles with a primary particle size of ~30 nm after aging for 2 h (Figure 3a,c), while retaining their amorphous state (Figure 6a). Meanwhile, the CaP particles immobilized CF, probably due to the electrostatic interactions between the ionized carboxyl groups in CF and the calcium ions in CaP [18,41]. Electrostatic interactions between the protonated piperazinyl groups in CF and the phosphate ions in CaP might also be involved in the CF immobilization in the CaP particles [42]. At this stage (2 h), approximately 27% of CF in the solution was immobilized in the CaP particles (Figure 5b). In the subsequent aging stage (from 2 to 24 h), the nearly spherical CaP particles grew further, immobilizing the residual CF in the solution and converting into irregularly shaped particles (Figure 3d). This morphological change was caused by the transformation of the CaP phase; the isotropic amorphous CaP phase was converted into anisotropic crystalline hydroxyapatite (Figure 6a). This phase transformation occurs spontaneously because hydroxyapatite is the most stable phase in a supersaturated environment with a nearly neutral pH [29,37] and accounts for the increased Ca/P elemental ratio in the long-aged particles (Figure 6b). In the final stage of aging (24 h), approximately 68% of CF in the solution was immobilized in the CaP particles (Figure 5b), resulting in an increased amount (~2.5 times in mg/tube) and content (~2 times in *w*/*w*%) of CF in the particles aged for 24 h (CF-CaP24h) compared with those aged for 2 h (CF-CaP2h).

Despite the higher CF content, CF-CaP24h released CF in the physiological salt solution more slowly than CF-CaP2h. A major cause of this difference was the lower solubility of crystalline hydroxyapatite in CF-CaP24h than that of amorphous CaP in CF-CaP2h under the tested conditions, as reported previously [30]. This suggests that the CF release from these particles is associated with the partial dissolution of their CaP matrix. The difference in the specific surface areas of CF-CaP2h and CF-CaP24h, as observed by TEM (Figure 3c,d), might also be involved in their different CF-release profiles.

Both CF-CaP2h and CF-CaP24h exhibited antibacterial activities against *S. mutans*, *A. naeslundii*, and *P. gingivalis* at higher doses (Figure 8). The dose–response relationship differed depending on the type of particles and bacteria. In the three doses tested in this study, the minimum effective dose of CF-CaP24h was 0.01 *w*/*v*% (net CF dose of 8.5 μg/mL) for all the tested bacteria, whereas that of CF-CaP2h was 0.1 *w*/*v*% (net CF dose of 42 μg/mL) for *A. naeslundii* and 0.01 *w*/*v*% (net CF dose of 4.2 μg/mL) for the other two bacteria. These results are reasonable considering the minimal inhibitory concentration (MIC) of CF (1 µg/mL [22] and 4 µg/mL [21] for *S. mutans*, 3.9 μg/mL [25] and 0.063-4 μg/mL [26] for *A. naeslundii*, and 0.064–0.25 μg/mL [24] and 0.019 μg/mL [23] for *P. gingivalis*). At lower doses, the concentration of CF released from the particles in the bacterial suspension was likely to be below the MIC.

Despite the slower CF release in the physiological salt solution, CF-CaP24h exhibited a higher antibacterial activity against *S. mutans* and *A. naeslundii* than CF-CaP2h at the medium dose of 0.01 *w*/*v*% (Figure 8a,b). This might be due to the acidification of the bacterial suspension caused by these acid-producing bacteria (Figure 9a,b), which accelerates the dissolution of the CaP matrix, not only of amorphous CaP but also of hydroxyapatite [43]. The net CF dose in the bacterial suspension provided by CF-CaP24h was approximately double than that provided by CF-CaP2h, reflecting the higher CF content in the former. Therefore, CF-CaP24h can release a higher CF concentration than CF-CaP2h in an acidified bacterial suspension via accelerated CaP matrix dissolution. The dissolution of the CaP matrix may be responsible for the acid-neutralizing effect of these particles, as reported in our previous study [12]. At a medium dose (0.01 *w*/*v*%), CF-CaP24h exhibited an acid-neutralizing effect on *A. naeslundii*, whereas CF-CaP2h did not. This can be attributed to the difference in the CaP phase between the two particles, hydroxyapatite (with hydroxide) in CF-CaP24h and amorphous CaP (without hydroxide) in CF-CaP2h.

The degree of acidification of the bacterial suspension by *P. gingivalis* was much lower than that by *S. mutans* and *A. naeslundii* (Figure 9). The pH values of the suspension of *P. gingivalis* decreased from 7.1–7.2 to 6.6–6.8 after incubation for 24 h, irrespective of the type of particles and their doses. Under this pH range, the release profile of CF from the particles should be similar to that in the physiological salt solution (pH 7.4 at 37 °C). Therefore, CF-CaP2h would likely release more CF than CF-CaP24h in the *P. gingivalis* suspensions. This may account for the relatively stronger (in average value) antibacterial activity of CF-CaP2h than that of CF-CaP24h at medium (0.01 *w*/*v*%) and higher (0.1 *w*/*v*%) doses (Figure 8c).

Both CF-CaP2h and CF-CaP24h showed antibiofilm activity against *S. mutans* at higher doses (Figure 10). In the three doses tested in this study, the minimum effective dose was 0.01 *w*/*v*% for both CF-CaP2h (net CF dose of 4.2 μg/mL) and CF-CaP24h (net CF dose of 8.5 μg/mL) in the two assays. According to a previous report, the half-maximal inhibitory concentration of CF against biofilm formation by *S. mutans* is 3.9 μM (~1.3 μg/mL) [44], which is consistent with our results.

Overall, our data suggest that the CF-immobilized CaP particles have the potential to be used as dental disinfectants against oral bacteria. The prepared particles are small; hence, they may be delivered to narrow spaces, such as dental fissures, pits, and periodontal pockets, which are regions of predilection for caries and periodontal diseases. When these particles are delivered to these regions, they are expected to release CF and exhibit antibacterial and antibiofilm activities against oral bacteria, along with an acid-neutralizing effect. These particles would therefore be effective in the prevention and treatment of oral diseases including dental caries and periodontal disease. The release of CF from the particles is mediated by the dissolution of the CaP matrix into calcium and phosphate ions. Thus, these particles have a favorable secondary effect on tooth remineralization. 

Despite presenting some credible findings, there are several limitations to this study. First, the release of CF was assayed using a neutralized physiological salt solution, which differs from the actual intraoral conditions. Hence, the effects of oral components and changes in the conditions (temperature, pH, etc.) on the properties and efficacy of the particles should be investigated in future research. Second, neither the storage stability nor the intraoral stability of the particles has yet been examined, and the intraoral kinetics of the particles remains unknown. Further modifications to the particles might be required to improve their functionalities, such as water-dispersibility, stability, and retention at the target sites. More detailed in vitro and in vivo studies are also required to confirm the potential of the CF-immobilized CaP particles.

## 5. Conclusions

CF-immobilized CaP particles were fabricated via a coprecipitation process using a supersaturated CaP solution. As the aging time in the coprecipitation process increased from 2 to 24 h, the CaP phase in the resulting particles transformed from amorphous to low-crystalline hydroxyapatite, and their Ca/P elemental ratio, yield, and CF content increased. The particles aged for 24 h released CF in the physiological salt solution more slowly than those aged for 2 h. Both particles exhibited antibacterial and antibiofilm activities, along with an acid-neutralizing effect against *S. mutans*, *P. gingivalis*, and *A. naeslundii*, whose dose–response relationship was slightly different. Aging time in the coprecipitation process was identified as a controlling factor affecting the physiochemical properties of the resulting CF-immobilized CaP particles and their functionality as a dental disinfectant against oral bacteria.

## Data Availability

Data are contained within the article.

## References

[1] Li X., Liu Y., Yang X., Li C., Song Z. (2022). The oral microbiota: Community composition, influencing factors, pathogenesis, and interventions. Front. Microbiol..

[2] Matsumoto-Nakano M. (2018). Role of *Streptococcus mutans* surface proteins for biofilm formation. Jpn. Dent. Sci. Rev..

[3] Hussain M., Stover C.M., Dupont A. (2015). *P. gingivalis* in periodontal disease and atherosclerosis–Scenes of action for antimicrobial peptides and complement. Front. Immunol..

[4] Dige I., Raarup M.K., Nyengaard J.R., Kilian M., Nyvad B. (2009). *Actinomyces naeslundii* in initial dental biofilm formation. Microbiology.

[5] Rajeshwari H.R., Dhamecha D., Jagwani S., Rao M., Jadhav K., Shaikh S., Puzhankara L., Jalalpure S. (2019). Local drug delivery systems in the management of periodontitis: A scientific review. J. Control. Release.

[6] Bapat R.A., Joshi C.P., Bapat P., Chaubal T.V., Pandurangappa R., Jnanendrappa N., Gorain B., Khurana S., Kesharwani P. (2019). The use of nanoparticles as biomaterials in dentistry. Drug Discov. Today.

[7] Sokolova V., Epple M. (2021). Biological and medical applications of calcium phosphate nanoparticles. Chem. Eur. J..

[8] Dorozhkin S.V. (2010). Nanosized and nanocrystalline calcium orthophosphates. Acta Biomater..

[9] Zalite V., Lungevics J., Vecstaudza J., Stipniece L., Locs J. (2022). Nanosized calcium deficient hydroxyapatites for tooth enamel protection. J. Biomed. Mater. Res..

[10] Xu J., Shi H., Luo J., Yao H., Wang P., Li Z., Wei J. (2022). Advanced materials for enamel remineralization. Front. Bioeng. Biotechnol..

[11] Xie C., Lu H., Li W., Chen F.M., Zhao Y.M. (2012). The use of calcium phosphate-based biomaterials in implant dentistry. J. Mater. Sci. Mater. Med..

[12] Nakamura M., Oyane A., Shimizu Y., Miyata S., Saeki A., Miyaji H. (2016). Physicochemical fabrication of antibacterial calcium phosphate submicrospheres with dispersed silver nanoparticles via coprecipitation and photoreduction under laser irradiation. Acta Biomater..

[13] Cao G., Jiang Y., Chen F., Lu B., Tan S., Feng V., Qi S., He S., Xu Y., Chen X. (2020). Antibacterial silver-doped calcium phosphate synthesized by an enzymatic strategy for initial caries treatment. Ceram. Int..

[14] Yang M., Ren J., Zhang R. (2017). Novel gallium-doped amorphous calcium phosphate nanoparticles: Preparation, application and structure study. J. Non-Cryst. Solids.

[15] Chen X., Tang Q.L., Zhu Y.J., Zhu C.L., Feng X.P. (2012). Synthesis and antibacterial property of zinc loaded hydroxyapatite nanorods. Mater. Lett..

[16] Kovtun A., Kozlova D., Ganesan K., Biewald C., Seipold N., Gaengler P., Arnold W.H., Epple M. (2012). Chlorhexidine-loaded calcium phosphate nanoparticles for dental maintenance treatment: Combination of mineralising and antibacterial effects. RSC Adv..

[17] Ghosh S., Wu V., Pernal S., Uskoković V. (2016). Self-setting calcium phosphate cements with tunable antibiotic release rates for advanced antimicrobial applications. ACS Appl. Mater. Interfaces.

[18] Kumar G.S., Govindan R., Girija E.K. (2014). In situ synthesis, characterization and *in vitro* studies of ciprofloxacin loaded hydroxyapatite nanoparticles for the treatment of osteomyelitis. J. Mater. Chem. B.

[19] Sangeetha K., Ashok M., Girija E.K., Vidhya G., Vasugi G. (2018). Strontium and ciprofloxacin modified hydroxyapatites as functional grafts for bone prostheses. Ceram. Int..

[20] Sharma D., Patel R.P., Zaidi S.T.R., Sarker M.M.R., Lean Q.Y., Ming L.C. (2017). Interplay of the quality of ciprofloxacin and antibiotic resistance in developing countries. Front. Pharmacol..

[21] Carreira C.d.M., dos Santos S.S.F., Jorge A.O.C., Lage-Marques J.L. (2007). Antimicrobial effect of intracanal substances. J. Appl. Oral Sci..

[22] Chotitumnavee J., Parakaw T., Srisatjaluk R.L., Pruksaniyom C., Pisitpipattana S., Thanathipanont C., Amarasingh T., Tiankhum N., Chimchawee N., Ruangsawasdi N. (2019). In vitro evaluation of local antibiotic delivery via fibrin hydrogel. J. Dent. Sci..

[23] Nalawade T.M., Bhat K.G., Sogi S. (2016). Antimicrobial activity of endodontic medicaments and vehicles using agar well diffusion method on facultative and obligate anaerobes. Int. J. Clin. Pediatr. Dent..

[24] Eick S., Schmitt A., Sachse S., Schmidt K.H., Pfister W. (2004). In vitro antibacterial activity of fluoroquinolones against *Porphyromonas gingivalis* strains. J. Antimicrob. Chemother..

[25] Elshikh M., Moya-Ramiırez I., Moens H., Roelants S., Soetaert W., Marchant R., Banat I.M. (2017). Rhamnolipids and lactonic sophorolipids: Natural antimicrobial surfactants for oral hygiene. J. Appl. Microbiol..

[26] Barberis C., Budia M., Palombarani S., Rodriguez C.H., Ramírez M.S., Arias B., Bonofiglio L., Famiglietti A., Mollerach M., Almuzara M. (2017). Antimicrobial susceptibility of clinical isolates of *Actinomyces* and related genera reveals an unusual clindamycin resistance among *Actinomyces urogenitalis* strains. J. Glob. Antimicrob. Resist..

[27] Shubhra Q.T.H., Oyane A., Nakamura M., Puentes S., Marushima A., Tsurushima H. (2017). Rapid one-pot fabrication of magnetic calcium phosphate nanoparticles immobilizing DNA and iron oxide nanocrystals using injection solutions for magnetofection and magnetic targeting. Mater. Today Chem..

[28] Bigi A., Boanini E. (2018). Calcium phosphates as delivery systems for bisphosphonates. J. Funct. Biomater..

[29] Nakamura M., Bunryo W., Narazaki A., Oyane A. (2022). High immobilization efficiency of basic protein within heparin-immobilized calcium phosphate nanoparticles. Int. J. Mol. Sci..

[30] Kadkhodaie-Elyaderani A., de Lama-Odría M.d.C., Rivas M., Martínez-Rovira I., Yousef I., Puiggalí J., del Valle L.J. (2022). Medicated scaffolds prepared with hydroxyapatite/streptomycin nanoparticles encapsulated into polylactide microfibers. Int. J. Mol. Sci..

[31] Pal A., Oyane A., Nakamura M., Koga K., Nishida E., Miyaji H. (2024). Fluoride-incorporated apatite coating on collagen sponge as a carrier for basic fibroblast growth factor. Int. J. Mol. Sci..

[32] Nugrahani I., Tjengal B., Gusdinar T., Horikawa A., Uekusa H. (2020). A comprehensive study of a new 1.75 hydrate of ciprofloxacin salicylate: SCXRD structure determination, solid characterization, water stability, solubility, and dissolution study. Crystals.

[33] Sahoo S., Chakraborti C.K., Mishra S.C. (2011). Qualitative analysis of controlled release ciprofloxacin/carbopol 934 mucoadhesive suspension. J. Adv. Pharm. Technol. Res..

[34] Jin B., Liu Z., Shao C., Chen J., Liu L., Tang R., De Yoreo J.J. (2021). Phase transformation mechanism of amorphous calcium phosphate to hydroxyapatite investigated by liquid-cell transmission electron microscopy. Cryst. Growth Des..

[35] Manoj M., Mangalaraj D., Ponpandian N., Viswanatan C. (2015). Core–shell hydroxyapatite/Mg nanostructures: Surfactant free facile synthesis, characterization and their in vitro cell viability studies against leukaemia cancer cells (K562). RSC Adv..

[36] Kumar K.C.V., Subha T.J., Ahila K.G., Ravindran B., Chang S.W., Mahmoud A.H., Mohammed O.B., Rathi M.A. (2021). Spectral characterization of hydroxyapatite extracted from black sumatra and fighting cock bone samples: A comparative analysis. Saudi J. Biol. Sci..

[37] Dorozhkin S.V. (2021). Synthetic amorphous calcium phosphates (ACPs): Preparation, structure, properties, and biomedical applications. Biomater. Sci..

[38] Pleshko N., Boskey A., Mendelsohn R. (1991). Novel infrared spectroscopic method for the determination of crystallinity of hydroxyapatite minerals. Biophys. J..

[39] Tao J., De Yoreo J.J. (2013). FTIR and raman studies of structure and bonding in mineral and organic–mineral composites. Methods in Enzymology.

[40] Moreau J.L., Sun L., Chow L.C., Xu H.H.K. (2011). Mechanical and acid neutralizing properties and bacteria inhibition of amorphous calcium phosphate dental nanocomposite. J. Biomed Mater. Res. Part B Appl. Biomater..

[41] Nardecchia S., Gutierrez M.C., Serrano M.C., Dentini M., Barbetta A., Ferrer M.L., del Monte F. (2012). In situ precipitation of amorphous calcium phosphate and ciprofloxacin crystals during the formation of chitosan hydrogels and its application for drug delivery purposes. Langmuir.

[42] Ikawa N., Kimura T., Oumi Y., Sano T. (2009). Amino acid containing amorphous calcium phosphates and the rapid transformation into apatite. J. Mater. Chem..

[43] Astasov-Frauenhoffer M., Varenganayil M.M., Decho A.W., Waltimo T., Braissant O. (2017). Exopolysaccharides regulate calcium flow in cariogenic biofilms. PLoS ONE.

[44] Mekky A.E.M., Sanad S.M.H. (2020). Novel bis(pyrazole-benzofuran) hybrids possessing piperazine linker: Synthesis of potent bacterial biofilm and MurB inhibitors. Bioorg. Chem..

